# Phosphorylation of transglutaminase 2 (TG2) at serine-216 has a role in TG2 mediated activation of nuclear factor-kappa B and in the downregulation of PTEN

**DOI:** 10.1186/1471-2407-12-277

**Published:** 2012-07-03

**Authors:** Yi Wang, Sudharsana R Ande, Suresh Mishra

**Affiliations:** 1 Department of Internal Medicine, University of Manitoba, 843 JBRC/715 McDermot Avenue, Winnipeg, MB, R3E 3P4, Canada; 2 Department of Internal Medicine & Physiology, University of Manitoba, Winnipeg, Canada

**Keywords:** Protein kinase A, Mice embryonic fibroblast, Protein kinase B, Reporter-gene assay, FACS analysis, Real-time PCR

## Abstract

**Background:**

Transglutaminase 2 (TG2) and its phosphorylation have been consistently found to be upregulated in a number of cancer cell types. At the molecular level, TG2 has been associated with the activation of nuclear factor-kappa B (NF-κB), protein kinase B (PKB/Akt) and in the downregulation of phosphatase and tensin homologue deleted on chromosome 10 (PTEN). However, the underlying mechanism involved is not known. We have reported that protein kinase A (PKA) induced phosphorylation of TG2 at serine-216 (Ser^216^) regulates TG2 function and facilitates protein-protein interaction. However, the role of TG2 phosphorylation in the modulation of NF-κB, Akt and PTEN is not explored.

**Methods:**

In this study we have investigated the effect of TG2 phosphorylation on NF-κB, Akt and PTEN using embryonic fibroblasts derived from TG2 null mice (MEF^*tg2-/-*^) overexpressing native TG2 or mutant-TG2 (m-TG2) lacking Ser^216^ phosphorylation site with and without dibutyryl cyclic-AMP (db-cAMP) stimulation. Functional consequences on cell cycle and cell motility were determined by fluorescence activated cell sorting (FACS) analysis and cell migration assay respectively.

**Results:**

PKA activation in TG2 overexpressing MEF^*tg2-/-*^ cells resulted in an increased activation of NF-κB and Akt phosphorylation in comparison to empty vector transfected control cells as determined by the reporter-gene assay and immunoblot analysis respectively. These effects were not observed in MEF^*tg2-/-*^ cells overexpressing m-TG2. Similarly, a significant downregulation of PTEN at both, the mRNA and protein levels were found in cells overexpressing TG2 in comparison to empty vector control and m-TG2 transfected cells. Furthermore, Akt activation correlated with the simultaneous activation of NF-κB and a decrease in PTEN suggesting that the facilitatory effect of TG2 on Akt activation occurs in a PTEN-dependent manner. Similar results were found with MCF-7 and T-47D breast cancer cells overexpressing TG2 and m-TG2 further supporting the role of TG2 phosphorylation in NF-κB activation and in the downregulation of PTEN.

**Conclusions:**

Collectively, these data suggest that phosphorylation of TG2 at Ser^216^ plays a role in TG2 mediated activation of NF-κB, Akt and in the downregulation of PTEN. Blocking TG2 phosphorylation may provide a novel strategy to attenuate NF-κB activation and downregulation of PTEN in TG2 overexpressing cancers.

## Background

Transglutaminase 2 (TG2) is the most diverse and ubiquitous member of the TG family of enzymes that catalyze posttranslational modification of proteins by crosslinking proteins via ε-(γ-glutamyl)lysine isopeptide bonds or through incorporating primary amine at glutamine residues [1,2]. In addition, TG2 can bind and hydrolyze GTP and ATP, and functions as a G-protein in cell signaling processes [3,4]. The GTP binding and transamidation function of TG2 are inversely associated with each other [5,6]. Furthermore, we have reported that TG2 has intrinsic kinase activity and phosphorylates a number of proteins involved in cell proliferation and/or apoptosis [7-9]. Moreover, we have shown that TG2 undergoes phosphorylation in response to PKA activation and PKA induced phosphorylation of TG2 modulates TG function and facilitates protein-protein interaction [10].

TG2 has been found to be upregulated in a number of cancers including breast, ovarian, pancreatic, colon cancers and shown to confer resistance to chemotherapeutic drugs and promotes invasive potential of cancer cells [2,11-15]. In contrast, downregulation of TG2 by small interfering RNA or inhibition of TG2 activity by specific inhibitors has been shown to increase their sensitivity to chemo therapy-induced cell death and inhibition of cell migration [16-18]. At the molecular level TG2 constitutively activates prosurvival factors nuclear factor-kappa B (NF-κB) and focal adhesion kinase/protein kinase B (FAK/Akt) [19,20]. In addition, TG2 negatively regulates the tumor suppressor phosphatase and tensin homologue deleted on chromosome 10 (PTEN) [19]. However, the underlying mechanism involved remains obscure. Although a role for the crosslinking function of TG2 has been implicated initially [20], however, no careful investigations have been performed to make an unequivocal conclusion. Furthermore, emerging evidence suggests that transamidation function of TG2 is not involved in this process [2].

Posttranslational modification of proteins by phosphorylation plays a critical role in the regulation of protein functions and in protein-protein interactions [21]. For example, phosphorylation of NF-κB, PTEN and Akt is known to play a critical role in the functional regulation of these molecules [22-24]. Phosphoproteomic analysis of cancer signaling networks have consistently identified TG2 as a phosphoprotein in a number of cancer cell types [25,26]http://www.phosphosite.org/proteinAction.do?id=4135&showAllSites=true. However, the kinase responsible for TG2 phosphorylation and functional consequence of TG2 phosphorylation in cancer cell is not known. We have reported that TG2 undergoes phosphorylation at serine-216 (Ser^216^) residue in response to PKA activation [10]. In addition, we have shown that phosphorylation of TG2 facilitates protein-protein interaction; upregulates TG2 kinase activity and inhibits crosslinking activity [9,10]. Moreover, recently we have found that overexpression of phospho mutant forms of TG2 downregulates EGFR [11]. The cAMP-dependent PKA signaling is known to interact with NF-κB signaling and plays a crucial role in the pathogenesis of a number of NF-κB-related diseases [27]. It is possible that phosphorylation of TG2 by PKA or other upstream kinases play a role in TG2 mediated activation of NF-κB, Akt and in the downregulation of PTEN which in turn contribute to the invasive and chemoresistance potential of TG2 in cancer. In this study, we have investigated the functional impact of loss of TG2 phosphorylation at Ser^216^ on TG2 mediated modulation of NF-κB, PTEN and Akt in embryonic fibroblasts derived from TG2 null mice (MEF^*tg2-/-*^) and in breast cancer cells. Herein we report that phosphorylation of TG2 at Ser^216^ has an important role in TG2 mediated activation of NF-κB and in the downregulation of PTEN resulting into Akt activation.

## Results

### PKA induced phosphorylation of TG2 at Ser^216^ facilitates downregulation of PTEN in MEF^tg2-/-^ cells

**Figure 1:**
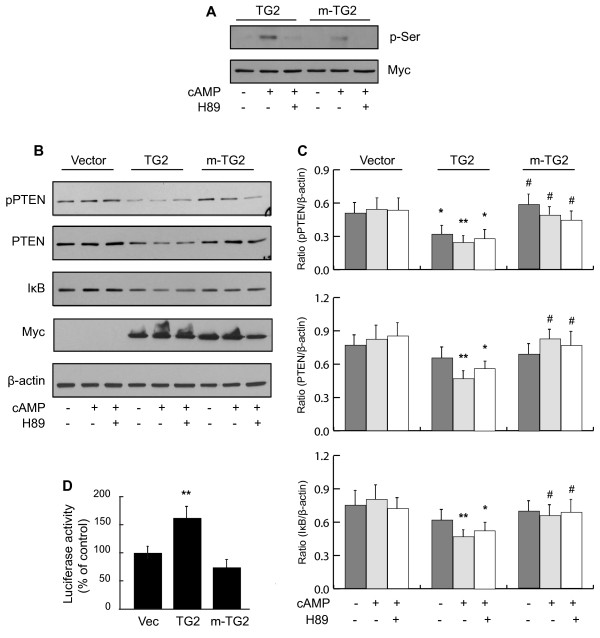
**PKA induced phosphorylation of TG2 at Ser**^**216 **^**facilitates downregulation of PTEN in MEF**^*tg2-/- *^**cells.** (**A**) Immunoblot showing Ser^216^ in TG2 as a predominant phosphorylation site in response to PKA activation by cAMP. MEF^*tg2-/-*^ cells were transfected with Myc tagged wild type TG2 and mutant-TG2 (lacking Ser^216^ phosphorylation site) constructs. 48 h post-transfection, cells were serum starved and incubated with or without db-cAMP (100 μM) in the presence or absence of a PKA inhibitor H89 (100 nM). Cells were harvested and cell lysates were analyzed by western immunoblotting using anti-phospho-serine and anti-Myc antibodies. Representative immunoblots of three different experiments are shown. (**B**) MEF^*tg2-/-*^ cells were transfected with various TG2 constructs. 48 h post-transfection, cells were serum starved and incubated with or without db-cAMP (100 μM) in the presence or absence of a PKA inhibitor H89 (100 nM). Cells were harvested and cell lysates were analyzed by western immunoblotting using protein and phospho-specific antibodies. Anti-Myc immunoblot showing expression levels of wild-type TG2 and m-TG2 is shown as a control. Representative immunoblots of four different experiments are shown. (**C**) Histograms showing relative quantification of protein/phospho-proteins as shown in left panel. (**D**) Histogram showing pNF-κB-MetLuc2-reporter gene activity in MEF^*tg-/-*^ cells transfected with TG2 and m-TG2 constructs. Data are represented as mean ± SEM, n = 4. * P < 0.05 and ** P < 0.01 (TG2 vs. empty vector control); # P < 0.05 (m-TG2 vs. TG2).

PKA that plays an important role in the pathogenesis of a number of NF-κB related diseases also phosphorylates TG2 at Ser^216^ residue [9,10,27]. PKA induced phosphorylation of TG2 at Ser^216^ was further confirmed using MEF^*tg-/-*^ cells overexpressing Myc tagged wild-type TG2 and Ser216Ala-TG2 mutant (m-TG2) lacking Ser^216^ phosphorylation site (Figure 1A). Since TG2 activates NF-κB, we explored whether PKA induced phosphorylation of TG2 at Ser^216^ play a role in TG2 mediated activation of NF-κB and subsequent downregulation of PTEN. To determine this, MEF^*tg2-/-*^ cells were transfected with Myc tagged wild-type TG2 and Ser216Ala-TG2 mutant (m-TG2). Expression level of wild-type TG2 and m-TG2 was confirmed by immunoblotting using anti-Myc antibody (Figure 1B). 48 h post-transfection, cells were incubated with db-cAMP in the presence and absence of a PKA specific inhibitor H89 and processed for further analysis [9,10]. Western immunoblot analysis revealed that overexpression of TG2 was sufficient to significantly downregulate PTEN (may be due to adequate basal PKA activity in these cells) in comparison to empty vector transfected control group (Figure 1B-C). Furthermore, db-cAMP stimulation led to further decrease in PTEN protein level that was partially protected in the presence H89 (Figure 1 B-C). On the contrary, m-TG2 fails to decrease PTEN protein levels even after db-cAMP induced PKA activation suggesting involvement of Ser^216^ in the downregulation of PTEN (Figure 1B-C). A similar change in the phosphorylation of PTEN was found using p-PTEN specific monoclonal antibody (Figure 1B-C). This antibody detects endogenous levels of PTEN only when phosphorylated at Ser^380^, Thr^382^ and Thr^383^. These phosphorylation sites are present at the carboxy-terminal noncatalytic regulatory domain, which regulates its stability and play an important role in the regulation of its biological activity [28,29]. Interestingly, an apparent difference in PTEN and pPTEN levels was found in cells expressing m-TG2 with and without db-cAMP treatment. This would indicate that in addition to Ser^216^, additional serine residue(s) in TG2 is also involved in this process and is consistent with the phosphorylation level of TG2 and m-TG2 observed in response to db-cAMP in MEF^*tg2-/-*^ cells (Figure 1A).

The NF-κB/Rel transcription factors are present in the cytosol in an inactive state associated with the inhibitory IκB proteins (20). Activation occurs via phosphorylation of IκB followed by proteasome-mediated degradation that results in the release and nuclear translocation of active NF-κB (20,22). As a prelude to NF-κB activation, we examined IκBα protein levels in cells overexpressing TG2 and m-TG2. IκBα was found to be significantly downregulated in TG2 overexpressing cells in comparison to empty vector transfected and m-TG2 overexpressing cells with db-cAMP stimulation (Figure 1B-C). No significant difference was found between m-TG2 overexpressing cells and empty vector transfected control group (Figure 1 B-C). To confirm that TG2 induced downregulation of PTEN is a consequence of NF-κB activation, we performed luciferase reporter-gene assay using a pNF-κB-MetLuc2-reporter. A significant upregulation of luciferase activity was observed in cells transfected with TG2 in comparison with empty vector transfected cells (Figure 1D). However, an increase in luciferase activity was not observed in m-TG2 transfected cells (Figure 1D). Most importantly, the luciferase activity inversely correlated with PTEN levels suggesting involvement of NF-κB in this process (Figure 1D). Collectively, these data suggest that phosphorylation of TG2 at Ser^216^ facilitates TG2 mediated activation of NF-κB and downregulation of PTEN.

### Phosphorylation of TG2 at Ser^216^ facilitates downregulation of PTEN in breast cancer cells

**Figure 2:**
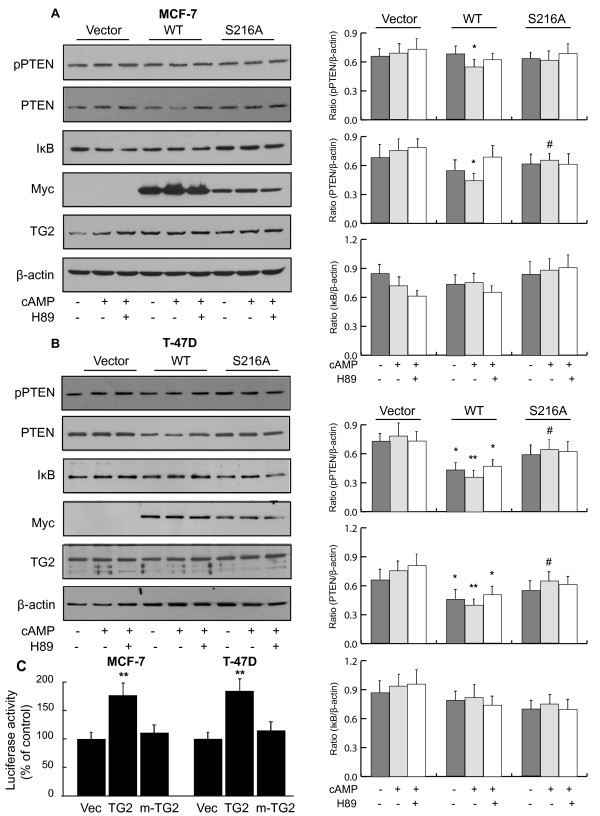
**PKA induced phosphorylation of TG2 at Ser**^**216 **^**facilitates downregulation of PTEN in breast cancer cells.** (**A**) MEF-7 and (**B**) T-47D breast cancer cells were transfected with various TG2 constructs. 48 h post-transfection, cells were serum starved and incubated with or without db-cAMP (100 μM) in the presence or absence of a PKA inhibitor H89 (100 nM). Cells were harvested and cell lysates were analyzed by western immunoblotting using protein and phospho-specific antibodies. Representative immunoblots of three different experiments are shown. Histograms showing relative quantification of protein/phospho-proteins are shown in right panel. (**C**) Histograms showing pNF-κB-MetLuc2-reporter gene activity in MCF-7 and T-47D cells transfected with TG2 and m-TG2 constructs. Data are represented as mean ± SEM, n = 3. * P < 0.05 (TG2 vs. vector control); ** P <0.01 (TG2 vs. vector control); # P < 0.05 (m-TG2 vs. TG2). WT, wild-type TG2; S216A, Ser216Ala-TG2 mutant.

To verify that TG2 induced activation of NF-κB and the downregulation of PTEN as observed in TG2 overexpressing MEF^*tg2-/-*^ is not cell specific and pertinent to TG2 protein, experiments were repeated using MCF-7 and T-47D breast cancer cells. A similar downregulation of PTEN protein level in MCF-7 and T-47D cells were found as in the case of MEF^*tg2-/-*^ cells overexpressing TG2 (Figure 2A-B). However, a differential effect on phospho-PTEN was observed in MCF-7 and T-47D in response to db-cAMP stimulation (Figure 2A-B). These differences may be attributed to differences in the prevailing signaling pathways in MCF-7 and T-47D cells that are known to target TG2. For example, TG2 has been identified as a target protein in EGFR signaling and EGFR expression level significantly differ in these two cell types [30,31]. Furthermore, similar to MEF^*tg2-/-*^ cells, pNF-κB-MetLuc2-reporter gene activity was upregulated in both cell lines overexpressing TG2 (Figure 2C). Taken together these data further confirms a role of TG2 phosphorylation in the activation of NF-κB and in the downregulation of PTEN.

### TG2 attenuates transcription of PTEN mRNA

**Figure 3:**
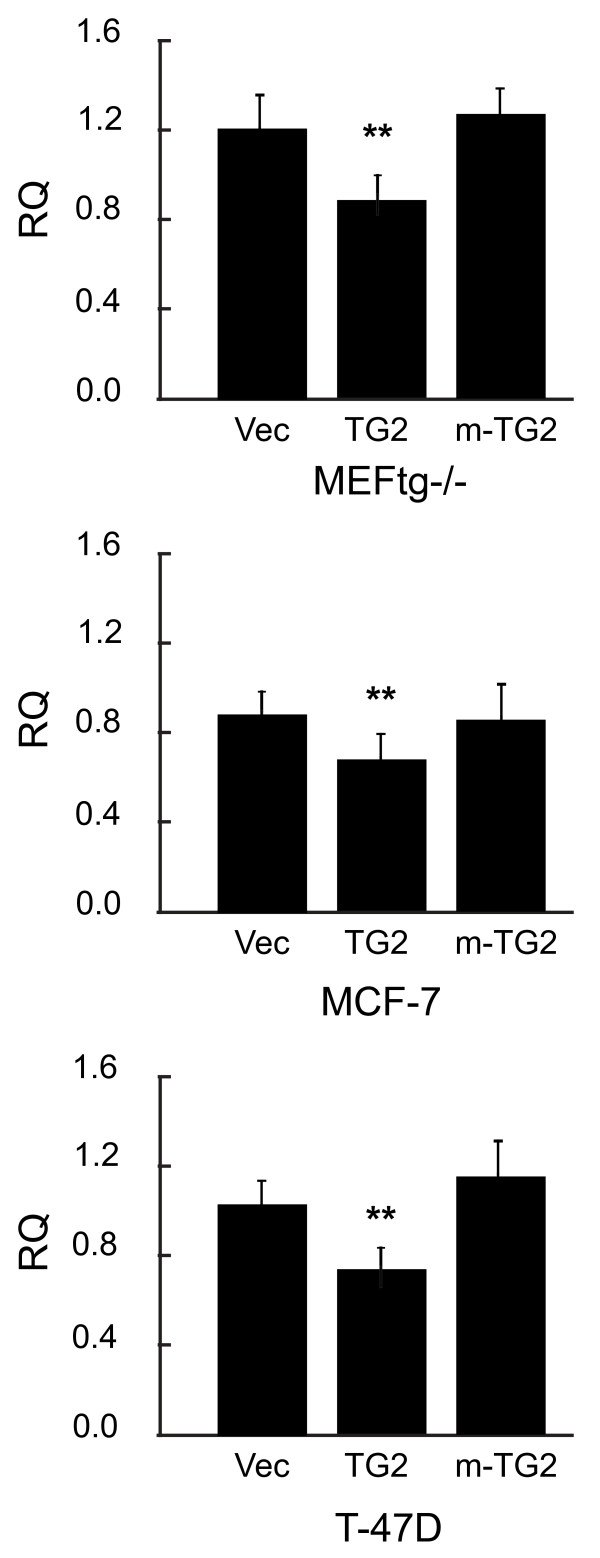
**PKA induced phosphorylation of TG2 at Ser**^**216 **^**facilitates downregulation of PTEN mRNA.** Histogram showing relative quantification (RQ) of PTEN mRNA expression levels as determined by real-time PCR in response to PKA activation by db-cAMP in MEF^*tg2-/-*^ and breast cancer cells in comparison to empty vector control group. The levels of mRNA were normalized against GAPDH mRNA. Cell transfection and treatments were performed as described in the figure legends 1 and 2. Data are represented as mean ± SEM, n = 4. **P < 0.01 (TG2 vs. vector control).

To determine whether TG2 mediated down regulation of PTEN occurs at the protein level or is a consequence of attenuated transcription, PTEN mRNA expression level was analyzed by real-time PCR in MEF^*tg2-/-*^, MCF-7 and T47D cells overexpressing TG2 and m-TG2. A significant decrease in PTEN mRNA expression was found only in cells overexpressing TG2 but not in m-TG2 overexpressing cells in comparison with empty vector transfected control group suggesting that TG2 mediated downregulation of PTEN occurs at the transcription level (Figure 3).

### TG2 mediated activation of NF-ÎºB and Akt are interconnected

**Figure 4:**
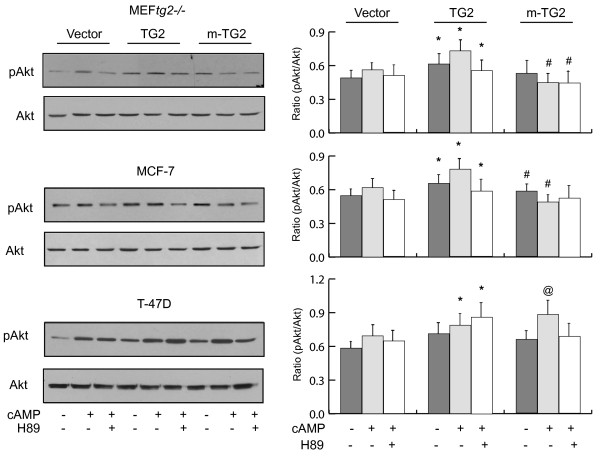
**TG2 mediated activation of NF-κB and Akt are interconnected.** Left panel: Representative immunoblots showing phosphorylation levels of Akt in MEF^*tg2-/-*^ and breast cancer cells overexpression TG2 and m-TG2 under the same experimental conditions as shown in Figure 1. Akt immunoblots are shown as controls. Right panel: Histograms showing quantitative difference in Akt phosphorylation as shown in the left panel. Data are represented as mean ± SEM, n = 4. * P < 0.05 (TG2 vs. vector control); # P < 0.05 (m-TG2 vs. TG2); @ P < 0.05 (m-TG2 vs. vector control).

TG2 has been implicated in the activation of Akt and PTEN is a major negative regulator of the phosphatidylinositol 3 kinase (PI3K)/Akt signaling pathway [19,32]. The main substrates of PTEN are inositol phospholipids generated by the activation of PI3K [33]. To investigate whether TG2 mediated activation of NF-κB and Akt are interconnected, we determined the phosphorylation status of Akt-Ser^473^, which is an important downstream substrate of PI3K and known to be upregulated in a number of cancers. In all three cell lines examined that is MEF^*tg2-/-*^, MCF-7 and T-47D, Akt phosphorylation was found to be increased in cells overexpressing TG2 in comparison with empty vector transfected control group (Figure 4). No difference in Akt phosphorylation was observed in cells overexpression m-TG2 in comparison with empty vector control group. However, a difference was apparent in Akt phosphorylation between cells overexpressing TG2 and m-TG2 which was significantly higher in TG2 overexpressing MEF^*tg2-/-*^ and MCF-7 cells with db-cAMP treatment (Figure 4). Unlike MEF^*tg2-/-*^ and MCF-7, an increase in Akt phosphorylation was observed in m-TG2 overexpressing T-47D cells in comparison with empty vector transfected control cells with db-cAMP stimulation. Most importantly, Akt activation directly correlated with the activation of NF-κB and inversely correlated with the downregulation of PTEN. Taken together, these data suggest that phospho-TG2 mediated activation of NF-κB and Akt are interconnected with each other and involves downregulation of PTEN.

### Overexpression of TG2 in MEF^tg2-/-^ enhances cell migration

**Figure 5:**
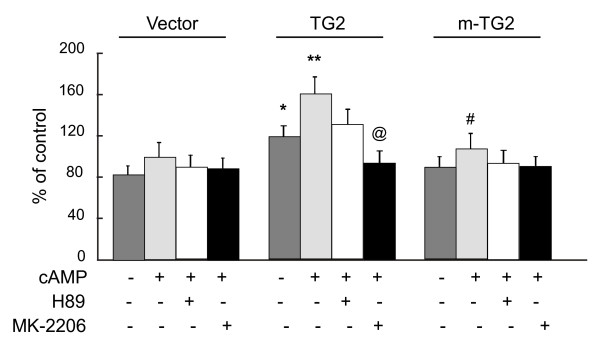
**Overexpression of TG2 in MEF**^***tg2-/- ***^**enhances cell migration.** The ability of TG2 and m-TG2 to induce cell migration was tested in response to db-cAMP (100 μM) stimulation in the presence of PKA inhibitor H89 (100 nM) and Akt inhibitor MK-2206 (20 nM). The data represent the mean ± SEM for n = 9; * P < 0.05 (TG2 vs. vector control); # P < 0.05 (m-TG2 vs. TG2); @ P < 0.05 (TG2 vs. Akt inhibitor).

To determine the functional consequence of the loss of phosphorylation at Ser^216^ in TG2, we studied the effect of overexpression of TG2 and m-TG2 on cell migration using a two chamber technique. Overexpression of TG2 alone led to significant increase in the migration of MEF^*tg2-/-*^ cells in comparison to empty vector transfected control cells which was further enhanced after db-cAMP stimulation (Figure 5). Furthermore, db-cAMP effect was significantly reduced in the presence of PKA inhibitor H89 suggesting a role of PKA in this process. On the contrary, m-TG2 overexpressing cells had no significant effect on cell migration in comparison with empty vector transfected control group even after db-cAMP treatment (Figure 5). However, a difference in cell migration was apparent between TG2 and m-TG2 in response to db-cAMP stimulation which was significantly lower in m-TG2 expressing cells suggesting a role of TG2 phosphorylation in TG2 mediated cell migration. Similarly, TG2 effect on cell migration was also inhibited by Akt inhibitor MK-2206 suggesting a role of Akt in TG2-induced cell migration (Figure 5).

### Cell cycle progression in MEF^tg2-/-^ and breast cancer cells

**Figure 6:**
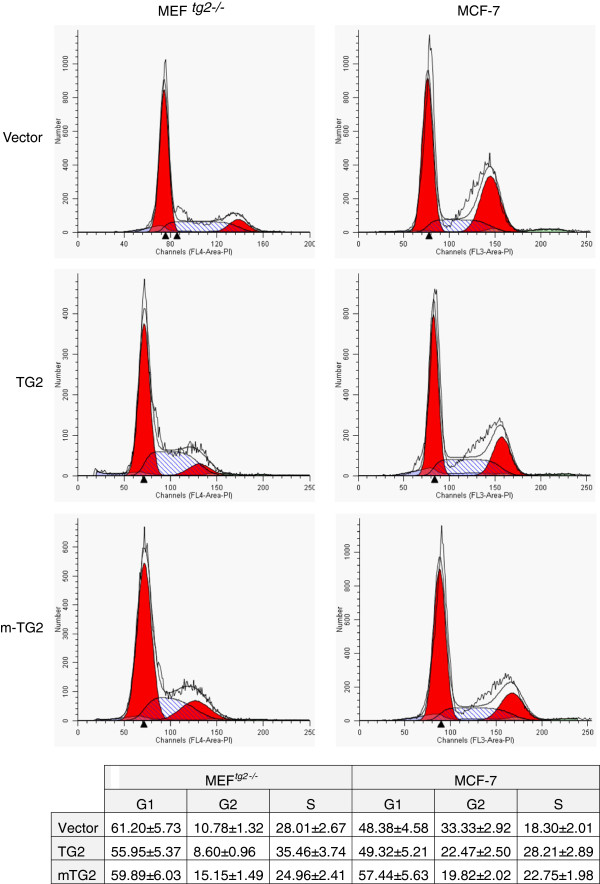
**Cell cycle progression in MEF**^***tg2-/- ***^**and MCF-7 breast cancer cells overexpressing TG2 and m-TG2.** Cell cycle distribution is shown in MEF^*tg2-/-*^ (left panel) and MCF-7 breast cancer cells (right panel). Cells at 60% confluency show an enhanced relative distribution in the S phase in TG2 overexpressing cells and a reduced relative distribution in the S phase in m-TG2 overexpressing cells (upper panels and table at bottom). The experiment was repeated three times. The table shows the mean ± SEM as a percentage for three independent experiments.

Akt and NF-κB activation is known to facilitate cell survival and cell proliferation in a number of cancers [34]. Since TG2 modulates phosphorylation of Akt at residues known to be involved in Akt activation, we evaluated the progression of MEF^*tg2-/-*^ and MCF-7 breast cancer cells overexpressing TG2 and m-TG2 through the cell cycle phases by FACS analysis. An increased progression of MEF^*tg2-/-*^ and MCF-7 cells through the S phase was found in TG2 overexpressing cells (Figure 6). In MEF^*tg2-/-*^ cells, an increase of ~27% was observed whereas in MCF-7 cells an increase of ~54% was found in comparison to empty vector transfected control group (Figure 6). However, a difference in the range of 30-38% was observed in m-TG2 overexpressing MEF^*tg2-/-*^ and MCF-7 cells through S phase in comparison with TG2 transfected cells, which was lower in m-TG2 overexpressing cells (Figure 6). Taken together, these data suggest that the loss of TG2 phosphorylation at Ser^216^ residue leads to significant reduction in cell progression through S phase.

## Discussion

PKA signaling plays an important role in the pathogenesis of a number of NF-κB related diseases including cancer and PKA activation confer resistance to trastuzumab in human breast cancer cells [27,35]. In patient samples, PKA signaling appeared to be enhanced in residual disease remaining after trastuzumab-containing neoadjuvant therapy [35]. Furthermore, in breast cancer cells the PKA signaling synergizes with NF-κB signaling [27,35]. PKA activating agents enhance NF-κB-dependent transcriptional activity whereas PKA inhibitors suppress NF-κB-induced cell proliferation and multiple NF-κB-dependent anti-apoptotic gene expression [27,35]. However, various signaling intermediates and protein substrates that mediate such effects remain to be identified. Previously, we have shown that PKA induced phosphorylation of TG2 at Ser^216^ regulates TG2 function and facilitates its interaction with phospho-serine/threonine binding protein 14-3-3 [10]. In this study, using MEF^*tg2-/-*^ cells overexpressing TG2 or m-TG2 (lacking Ser^216^ phosphorylation site), we unraveled a novel role of TG2 phosphorylation at Ser^216^ in the activation of NF-κB, Akt and in the downregulation of PTEN. We provide evidence that phosphorylation of TG2 at Ser^216^ facilitates TG2 mediated NF-κB activation, which induces downregulation of PTEN, resulting in Akt activation. As TG2 induced activation of Akt was found in the absence of receptor tyrosine kinase or PI3K stimulation, this would imply that TG2 induced activation of Akt under the experimental condition used in this study mainly occurs through downregulation of PTEN. Most importantly, the loss of phosphorylation at Ser^216^ in TG2 completely blocked TG2-induced effect on NF-κB activation and downregulation of PTEN mRNA as determined by reporter-gene assay and real-time PCR respectively.

Biochemically, PTEN is a phosphatase that dephosphorylates PIP3 that is produced by PI3K and function as a ubiquitous inhibitor of PI3K-dependent signaling [33,34]. The PI3K/PTEN signaling controls a large and diverse set of PIP3-binding proteins, the best characterized of which are the Akt protein kinases [34]. In this way, PI3K and PTEN orchestrate cell responses to growth factors, cytokines, integrins and other intercellular mediators and contribute to the growth, motility, survival and metabolic responses of many cell types [34]. Our data indicates that phosphorylation of TG2 may facilitate integration of factors into NF-κB signaling cascade and contribute to the growth, survival and motility of cancer cells as reported earlier [1,2]. In this context it should be noted that the TG2 promoter contains functional NF-κB binding elements [36,37]. It is plausible that TG2 mediated activation of NF-κB might payback by upregulation of TG2, and create a feed-forward loop involving phospho-TG2-NF-κB-TG2 leading to sustained inhibition of PTEN and constitutive activation of Akt in cancers characterized by TG2 upregulation along with constitutive activation of NF-κB.

The motif containing Ser^216^ phosphorylation site partially overlaps with a functional BH3-only motif identified in TG2 [38]. The BH3 motif identified in TG2 facilitates interaction of TG2 with the pro-apoptotic Bcl-2 family member Bax but not with anti-apoptotic members Bcl-2 and Bcl-X_L_[38]. In this context it should be noted that the phosphorylation of a specific serine residue in BH3 domain of BAD mediates its interaction with Bcl-X_L_ and increases the accessibility of another serine residue within BH3 domain to PKA induced phosphorylation [39]. The motif containing Ser^216^ in TG2 contains additional serine residue (i.e., Ser^212^) which is also present as a PKA consensus phosphorylation site (^209^RDCSRRSSPVYVGRV^223^). It is possible that phosphorylation of TG2 at Ser^216^ plays a similar role in its interaction with Bcl-2 family members which in turn may attenuate interaction of TG2 with pro-apoptotic members or facilitate interaction with anti-apoptotic members of Bcl-2 family of proteins. Furthermore, we have reported that phosphorylation of TG2 at Ser^216^ facilitates its interaction with protein 14-3-3, a phospho-serine/threonine binding protein [10]. Moreover, 14-3-3 protein is known to target BH3-only protein and emerging evidence suggests that 14-3-3 proteins are involved in cancer development [40]. It is conceivable that cancer promoting function of TG2 may also be mediated through phosphorylation-dependent interaction with 14-3-3 and Bcl-2 family of proteins that warrants further investigation.

**Figure 7:**
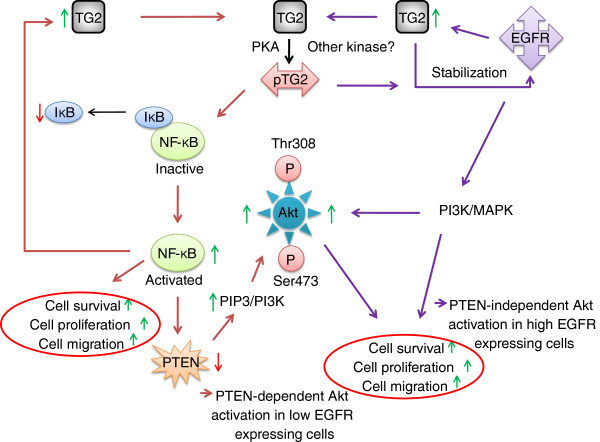
**Schematic diagram showing potential mechanism involved in tumor promoting function of TG2.** We propose that depending on upstream stimuli TG2 contribute to Akt activation in a PTEN-dependent and -independent manner.

As TG2 has been identified as a phospho-protein in multiple cancer cells/tissues, this would imply that TG2 may serve as a target protein for a number of upstream kinases and mediate the effect of tumor promoting factors. For instance, although we have identified Ser^216^ as a PKA phosphorylation site, the phosphorylation of Ser^216^ by other kinases may not be ruled out. This may also explain the differences observed in Akt phosphorylation and pPTEN/PTEN levels in three different cell lines used in this study. For example, TG2 has been identified as a downstream target of EGFR, and EGFR expression level is known to significantly vary in various breast cancer cell lines including MCF-7 and T-47D cells [41]. Moreover, recently we have reported that TG2 attenuates ligand-dependent downregulation of EGFR in EGFR expressing cells and overexpression of phospho mutant forms of TG2 downregulates EGFR [11]. Analysis of TG2 motif spanning Ser^216^ with phospho-motif servers ( http://networkin.info/version_2_0/search.php and http://www.hprd.org) predicted Ser^212^ and Ser^216^ as potential sites of phosphorylation by a number of kinases known to be involved in tumorigenic signals such as calmodulin-dependent protein kinase II, casein kinase II, MAPK, PKC, RSK and cdk5. In addition to Ser^216^, phosphorylation of TG2 at Tyr^219^ and Tyr^369^ has been identified in a number of cancer cells [25,26]. It is possible that phosphorylation of TG2 facilitates integration of multiple signaling pathways to NF-κB and PI3K/Akt signaling and facilitate tumorogenesis. Furthermore, this would imply that depending on upstream stimuli TG2 may contribute to Akt activation in a PTEN-dependent and -independent manner (Figure 7).

## Conclusions

In summary, data presented here provides new insight into the underlying mechanism involved in TG2 mediated activation of NF-κB, Akt and in the downregulation of PTEN that have been implicated in the invasive and chemoresistant potential of cancers. Further studies to identify the factors that are involved in the regulation of TG2 phosphorylation at Ser^216^ and other potentially important phosphorylation sites will hopefully provide information for the development of novel strategies to reduce the enhanced cancer growth associated with TG2 upregulation. Continued research will determine if interfering with the phosphorylation of TG2 has preventive or therapeutic application in cancer.

## Methods

### Reagents

MEF^*tg2*-/-^ cells were obtained from TG2 null mice [9,10]. MCF-7 and T47D breast cancer cells were obtained from American Type Culture Collection (Danvers, MA) and cell culture reagents and fetal bovine serum from Invitrogen (Carlsbad, CA). Akt (#9916) and PTEN (#9652) sampler kit and anti-Myc (#2272) antibodies were purchased from Cell Signaling Technology (Danvers, MA) and HRP-conjugated secondary antibodies were obtained from Santa Cruz Biotechnology (Santa Cruz, CA) and enhanced chemiluminescence (ECL) reagents from Promega (Madison, WI). Other reagents were purchased from Sigma-Aldrich (Oakville, ON) or as otherwise stated.

### Animals

TG2 knockout C57BL/6 mice were generously provided by Dr. Nikolaos Frangogiannis (Baylor College of Medicine, Houston, Texas, USA) with permission from Dr. Gerry Melino (University of Leicester, UK). Experiments involving mice were performed as approved by the Animal Care Committee of the University of Manitoba.

### Cell culture and transfection

MEF and breast cancer cell culture and treatments were performed as described before [9,10]. The pCMV vector containing Myc tagged human TG2 (Myc-TG2, Origene Technology, USA) was used to generate TG2 mutant. Ser216Ala-TG2 mutant lacking Ser^216^ phosphorylation sites was made by a site-directed mutagenesis kit using two complementary nucleotide primers (forward, 5' CTCCCGCCGCAGCGCCCCCGTCTACGTG 3' and reverse, 5' CACGTAGACGGGGGCGCTGCGGCGGGAG 3') containing desired mutation and Myc-TG2 as the template. Authenticity of all constructs was confirmed by DNA sequencing. Transfections with various constructs were performed using FuGENE HD transfection reagent (Roche, Germany) according to the manufacturer’s instructions. Cells treatments were performed 48 h post-transfection. Cell culture medium was exchanged for serum free media, and cells were serum starved for 6 h. Subsequently cells were treated with db-cAMP (100 μM) for 1 h or as otherwise stated in the presence and absence of PKA inhibitor H89 (100 nM).

### Western blotting

Cell lysates were prepared using lysis buffer (Sigma-Aldrich), containing protease inhibitor cocktail as described previously [9]. Protein concentrations of lysates were determined by the Bradford protein assay with bovine serum albumin (BSA) as the standard. Proteins were resolved on 10% SDS-PAGE and transferred onto nitrocellulose membranes. Membranes were blocked in 5% blocking grade milk and processed for incubation with primary and HRP-conjugated secondary antibodies [9,10]. Protein band was visualized using ECL and Kodak film.

### Real-time PCR

Total RNA was extracted using RNA easy plus mini kit (Qiagen, Canada) from MEF^*tg-/-*^ and breast cancer cells transfected with empty vector, wild type TG2 and mutant-TG2 (m-TG2, lacking Ser^216^ phosphorylation site) constructs. The cDNA was synthesized from total RNA (1 μg) using reverse transcriptase and oligo-deoxythymidine primers [42]. The following primers were used for amplification of cDNA using real-time PCR: PTEN, forward 5’-AAGACCATAACCCACCACAGC-3’ and reverse 5’-TCATTACACCAGTTCGTCCCT-3’; GAPDH, forward 5’-CATCACCATCTTCCAGGAGCG-3’ and reverse 5’-TGACCTTGCCCACAGCCTTG-3’. Amplification conditions used were 40 cycles of denaturation for 30 s at 94°C, 30 s annealing at 50°C, and 30 s extension at 72°C.

### FACS analysis

Cell cycle analysis was performed by fluorescence activated cell sorting (FACS) as described before [9]. In brief, MEF^*tg-/-*^ cells were transfected with various TG2 plasmids for 48 hours. Subsequently cells were collected by trypsinization, pelleted at 800 x *g* for 10 min, and fixed in 70% ethanol. DNA content was evaluated by flow cytometry with propidium iodide (PI) staining.

### Cell motility assay

MEF^*tg2-/-*^ cells were used for the cell motility assays. Polycarbonate filters (Thermo scientific, 8 um pore size) with cells which were transfected with various TG2 constructs were placed in wells of 6-well plates. Incubation was carried out with and without db-cAMP (100 μM) in the presence and absence of PKA inhibitor H89 (100 nM). The filters were removed and fixed in 1:10 diluted formalin for 1 h at RT. Cells on upper filter surface were removed carefully with cotton swab. The filters were stained in hematoxylin for 10 min, and cells on the lower surface of the filter were counted under a light microscope [43].

### Ready-To-Glow secreted luciferase reporter assay

MEF^*tg2-/-*^ and breast cancer cells were co-transfected with TG2 and pNFkB-MetLuc2-Reporter (Clontech, Mountain View, CA) constructs as per manufacturer’s protocol. Subsequently, cells were serum-starved for 6 hours, treated with 100 μM db cAMP for 1.5 h. Samples of supernatant media (100 μL) were collected. Subsequently 10 μL of 1 x substrate/reaction buffer was added to each sample and activity was measured using a Luminometer (Lumat LB 9507). The transfection efficiency was normalized by adding substrate buffer to cell lysates of each sample and light signals were recorded using a Luminometer [42].

### Statistical analysis

Experimental results are shown as means ± SEM. One-way ANOVA with Dunnett’s test was used for multiple comparisons. P values <0.05 were considered significant difference.

## Abbreviations

Akt: Protein kinase B/Akt; Ala: Alanine; ATP: Adenine nucleotide triphosphate; BSA: Bovine serum albumin; cdk5: Cyclin-dependent kinase 5; db cAMP: Dibutyryl cyclic adenosine monophosphate; ECL: Enhanced chemiluminescence; EGFR: Epidermal growth factor receptor; FACS: Fluorescence activated cell sorting; GTP: Guanine nucleotide triphosphate; HRP: Horse radish-peroxidase; MAPK: Mitogen activated protein kinase; MEF^*tg2-/-*^: Mice embryonic fibroblast derived from TG2 null mice; m-TG2: Mutant-transglutaminase 2; NF-κB: Nuclear factor-kappa B; PAkt: Phosphorylated Akt; PCR: Polymerase chain reaction; PI: Propidium iodide; PI3K: Phosphatidylinositol 3 kinase; PIP3: Phosphatidylinositol triphosphate; PKA: Protein kinase A; PKC: Protein kinase C; PTEN: Phosphatase and tensin homologue deleted on chromosome 10; PPTEN: Phosphorylated PTEN; RQ: Relative quantification; RSK: Ribosomal S6 kinase; SDS-PAGE: Sodium dodecyl polyacrylamide gel electrophoresis; SEM: Standard error of mean; Ser: Serine; S phase: Synthesis phase; TG2: Transglutaminase 2; Thr: Threonine; Tyr: Tyrosine.

## Competing interests

The authors declare that they have no competing interests.

## Authors' contributions

YW and SRA performed the experiments and help in design of the experiments and data analysis, SM participated in design of the experiments, data analysis and wrote the manuscript. All authors read and approved the final manuscript.

## Pre-publication history

The pre-publication history for this paper can be accessed here:

http://www.biomedcentral.com/1471-2407/12/277/prepub
